# Supplementary material for: Genetic and Environmental Factors Co-Contributing to Behavioral Abnormalities in adnp/adnp2 Mutant Zebrafish
Source: Int J Mol Sci. 2024 Aug 30;25(17):9469. doi: 10.3390/ijms25179469 (PMC11395604; doi:10.3390/ijms25179469)
Supplement: Supplementary file 1 [file ijms-25-09469-s001.zip › ijms-3174355-supplementary.pdf]

Figure captions

Supplementary figure S1. Related to Figure 1.

Supplementary figure S2. Related to Figure 2.

Supplementary figure S3. Related to Figure 3.

Supplementary figure S4. Related to Figure 4.

Supplementary figure S5. Related to figure 5.

Supplementary figure S6. Related to Figure 7.

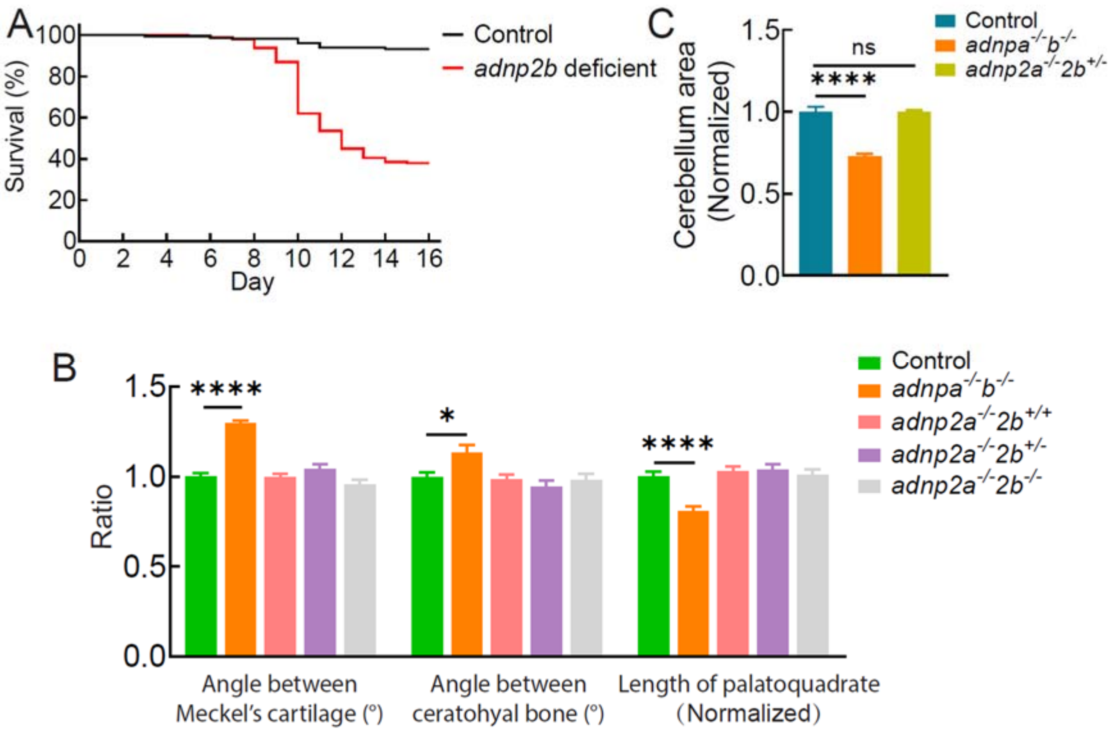

Supplementary figure S1. Related to Figure 1. (A) Death curve of *adnp2b* deficient (*adnp2b* deficient indicated *adnp2b*$^{+/-}$ in-cross) offspring within 16 days. (B) Histogram of angle between Meckel's cartilage, angle between ceratohyal bone, length of palatoquadrate (C) Relative cerebellar area of control, *adnpa*$^{-/-}$; *adnpb*$^{-/-}$ and *adnp2a*$^{-/-}$; *adnp2b*$^{+/-}$ adults at 4 months old. Data are presented as mean ± SEM; *P < 0.05,

****P < 0.0001. ns represents no significance.

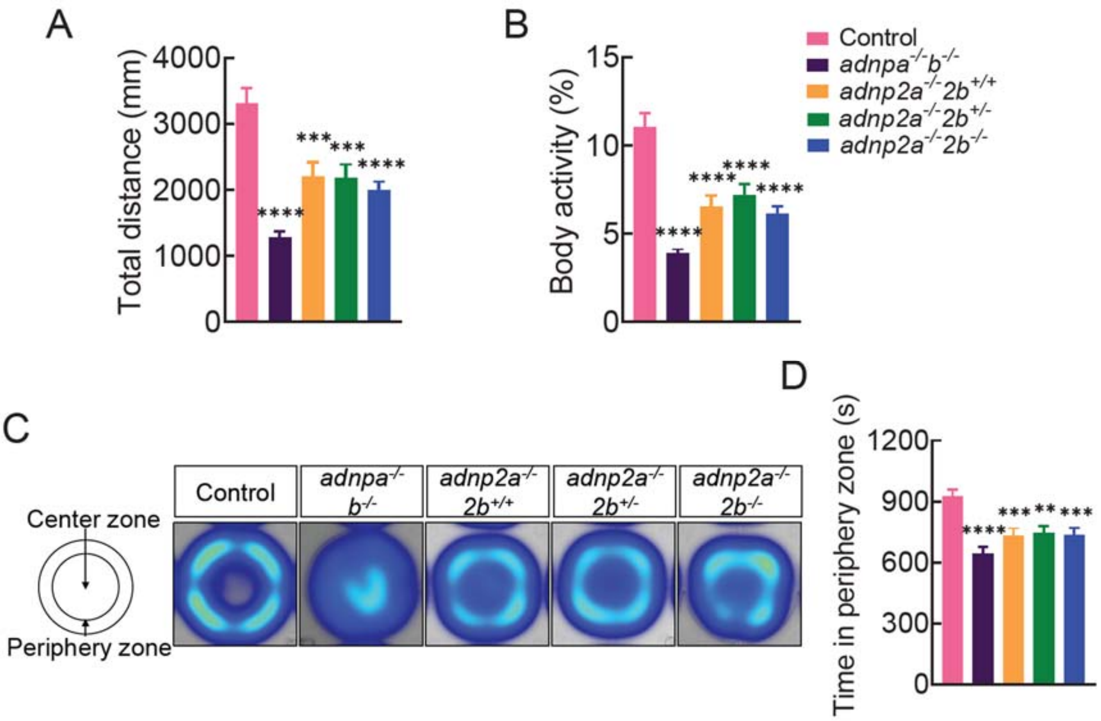

Supplementary figure S2. Related to Figure 2. (A) Graph showing the total distance (millimeters) travelled of the indicated groups. (B) Graph showing the body activity of the indicated groups. (C) Heat map visualization of larvae in the indicated groups. (D) Graph showing the time (second) spent in the peripheral zone of the indicated groups. Data are presented as mean± SEM; **P <0.01, ***P < 0.001, ****P < 0.0001. ns represents no significance.

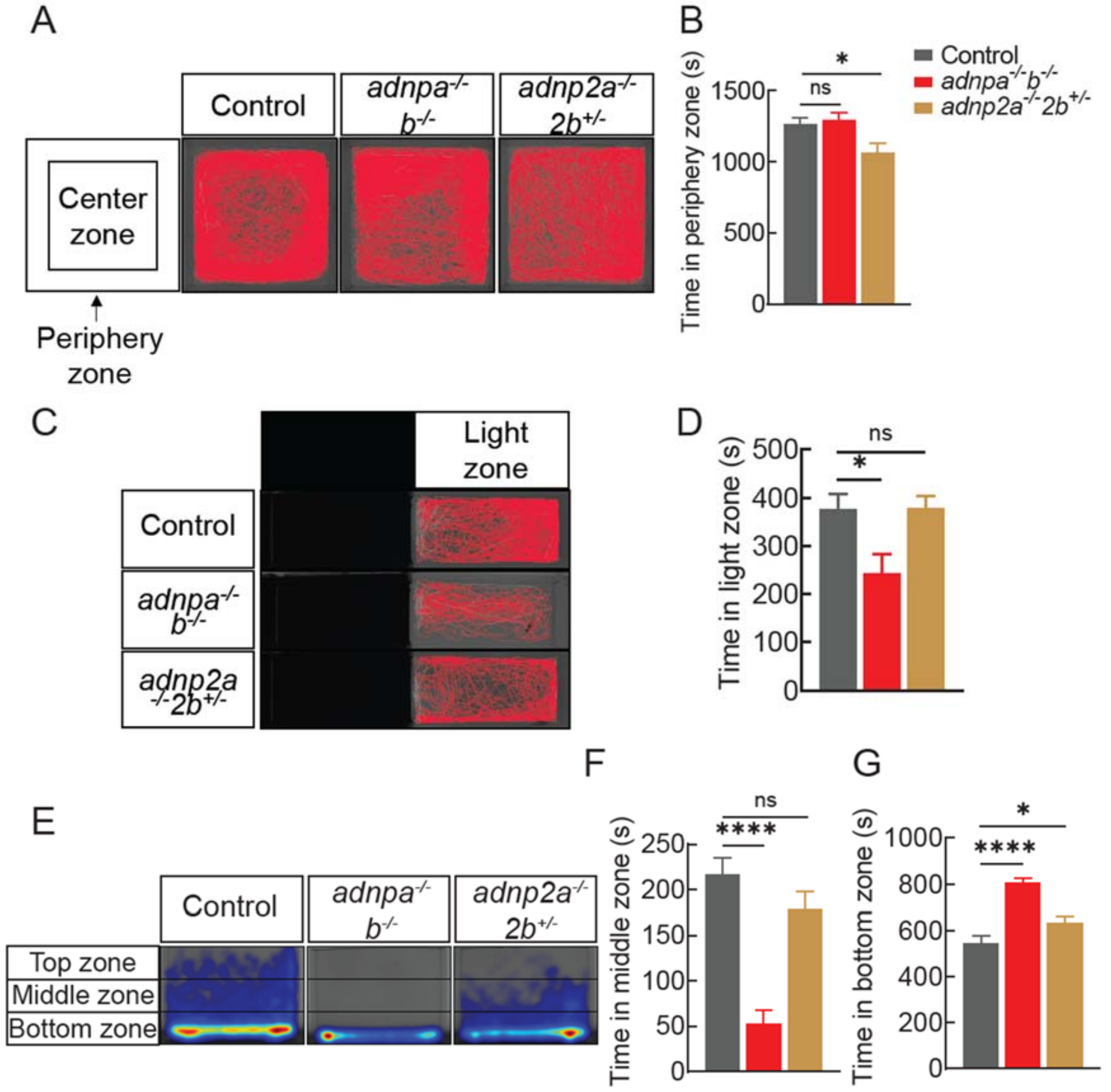

Supplementary figure S3. Related to Figure 3. (A) Swimming trajectory traces of control, *adnpa*$^{-/-}$; *adnpb*$^{-/-}$, and *adnp2a*$^{-/-}$; *adnp2b*$^{+/-}$ adults in thigmotaxis test (n=28 per group). The central area is equal to the peripheral area. (B) Graph showed the time (second) in peripheral zone of control, *adnpa*$^{-/-}$; *adnpb*$^{-/-}$, and *adnp2a*$^{-/-}$; *adnp2b*$^{+/-}$ fish (n=26 per group). (C) Trajectory traces of control, *adnpa*$^{-/-}$; *adnpb*$^{-/-}$, and *adnp2a*$^{-/-}$; *adnp2b*$^{+/-}$ adults in light/dark box test. (D) Graph showed the time in light zone of control, *adnpa*$^{-/-}$; *adnpb*$^{-/-}$, and *adnp2a*$^{-/-}$; *adnp2b*$^{+/-}$ adults in light/dark test. (E) Swimming trajectory traces of control, *adnpa*$^{-/-}$; *adnpb*$^{-/-}$, and *adnp2a*$^{-/-}$; *adnp2b*$^{+/-}$ adults in novel tank test (n=25 per group). (F-G) Graph showed the time (second) in

middle and bottom zones of control, *adnpa*$^{-/-}$; *adnpb*$^{-/-}$, and *adnp2a*$^{-/-}$; *adnp2b*$^{+/-}$ adults. Data are presented as mean ± SEM; *P < 0.05, ****P < 0.0001. ns represents no significance.

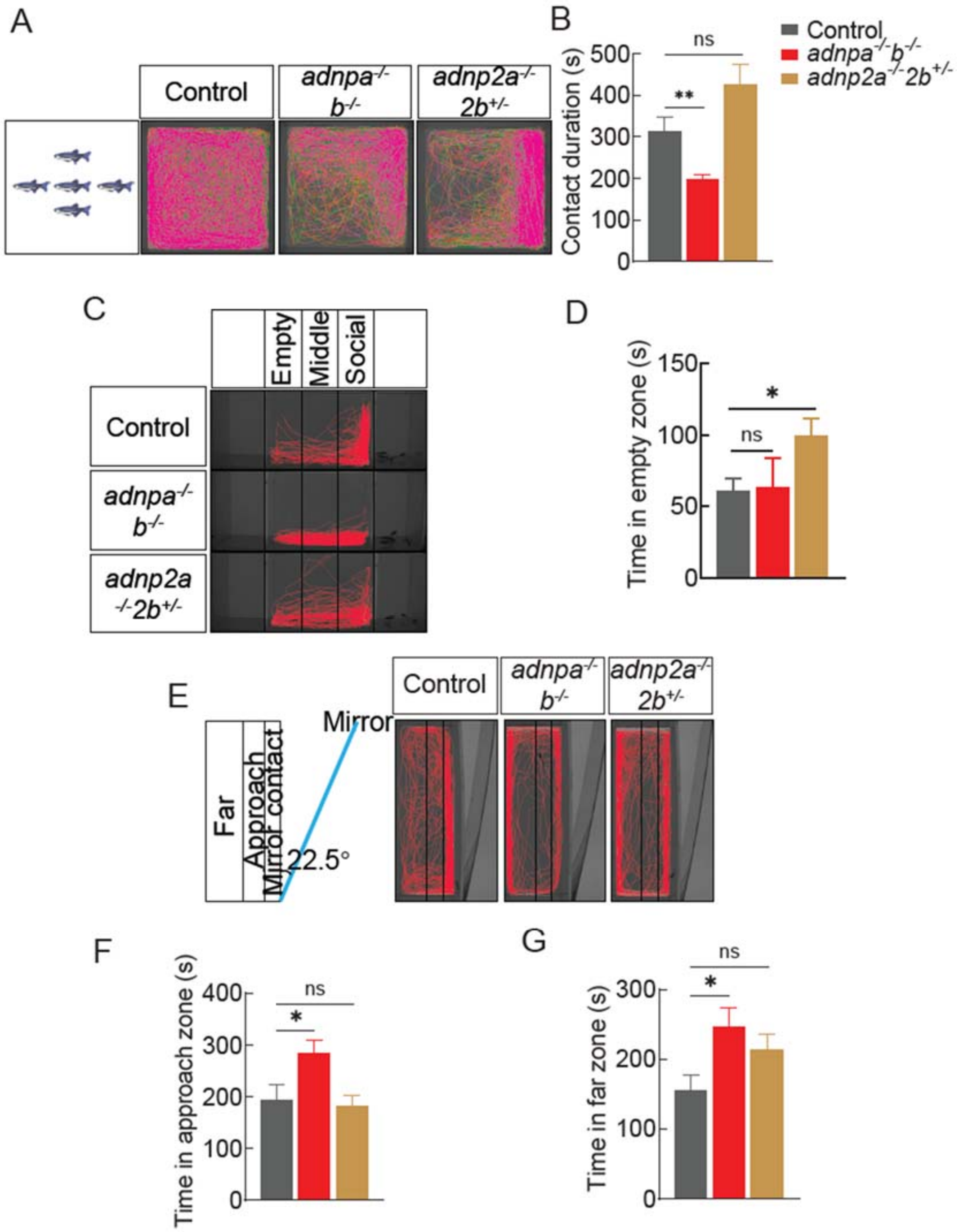

Supplementary figure S4. Related to Figure 4. (A) Trajectory traces of control, *adnpa*$^{-/-}$; *adnpb*$^{-/-}$, and *adnp2a*$^{-/-}$; *adnp2b*$^{+/-}$ adults in shoaling test. (B) Graph showed

contact time of the larvae from control, *adnpa*$^{-/-}$; *adnpb*$^{-/-}$, and *adnp2a*$^{-/-}$; *adnp2b*$^{+/-}$ adults. (C) Swimming trajectory traces of control, *adnpa*$^{-/-}$; *adnpb*$^{-/-}$, and *adnp2a*$^{-/-}$; *adnp2b*$^{+/-}$ adults in three-tank test. (D) Graph showed the time in empty zone of control, *adnpa*$^{-/-}$; *adnpb*$^{-/-}$, and *adnp2a*$^{-/-}$; *adnp2b*$^{+/-}$ adults. (E) Trajectory traces of control, *adnpa*$^{-/-}$; *adnpb*$^{-/-}$, and *adnp2a*$^{-/-}$; *adnp2b*$^{+/-}$ adults in mirror test. (F-G) Graph showed the time (second) in far and approach zones. Data are presented as mean± SEM; *P < 0.05, **P <0.01. ns represents no significance.

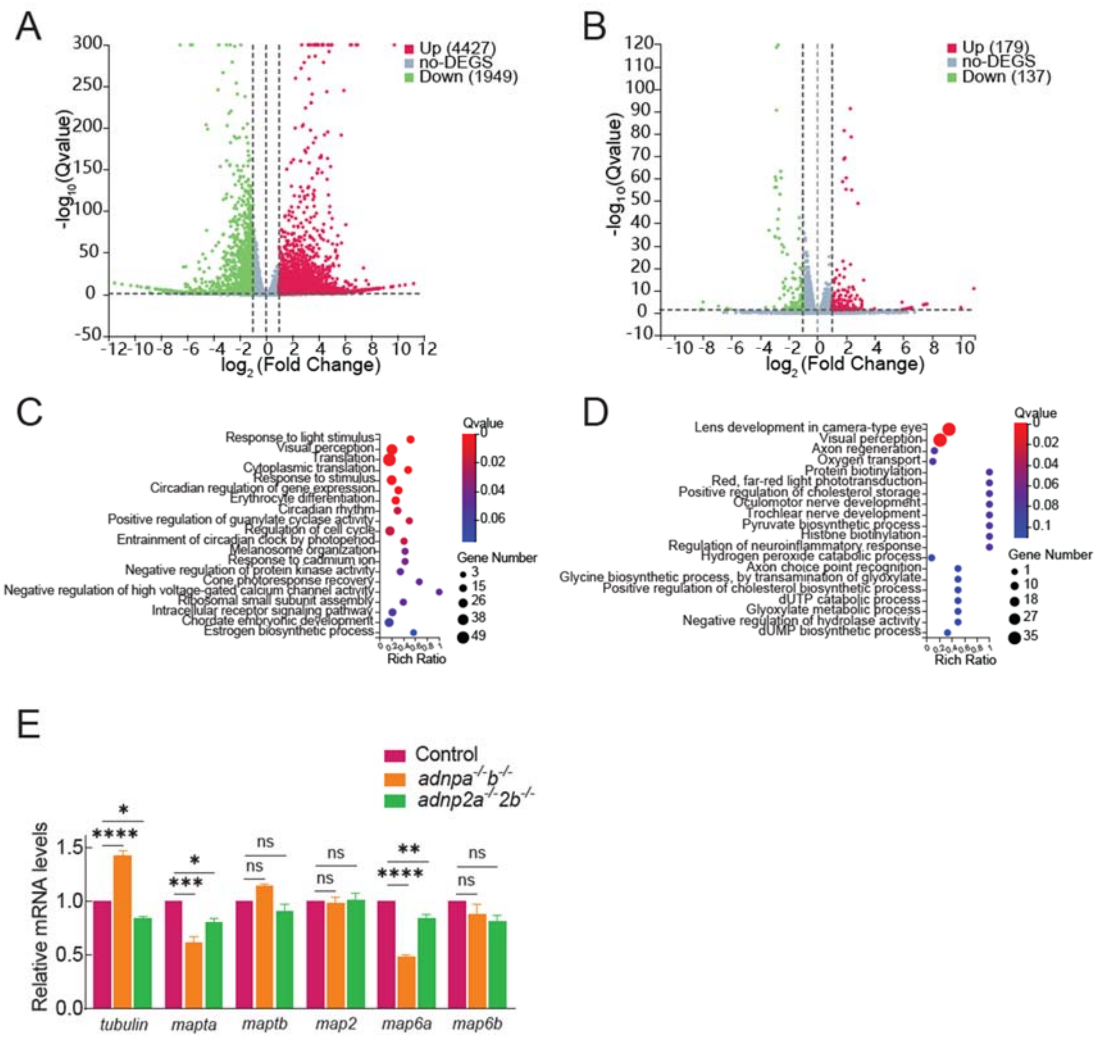

Supplementary figure S5. Related to figure 5. (A) Volcano plot showed the up- and down-regulated DEGs between control and *adnpa*$^{-/-}$; *adnpb*$^{-/-}$ double mutants. (B)

Volcano plot showed the up- and down-regulated DEGs between control and *adnp2a*$^{-/-}$; *adnp2b*$^{-/-}$ double mutants. (C) GO results of down-regulated DEGs between control and *adnp* mutant at 7 dpf. (D) GO results of down-regulated DEGs between control and *adnp2* mutant. (E) The qRT-PCR results of microtubule-related genes. Data are presented as mean ± SEM; **P* < 0.05, ***P* < 0.01, ****P* < 0.001, *****P* < 0.0001. ns represents no significance.

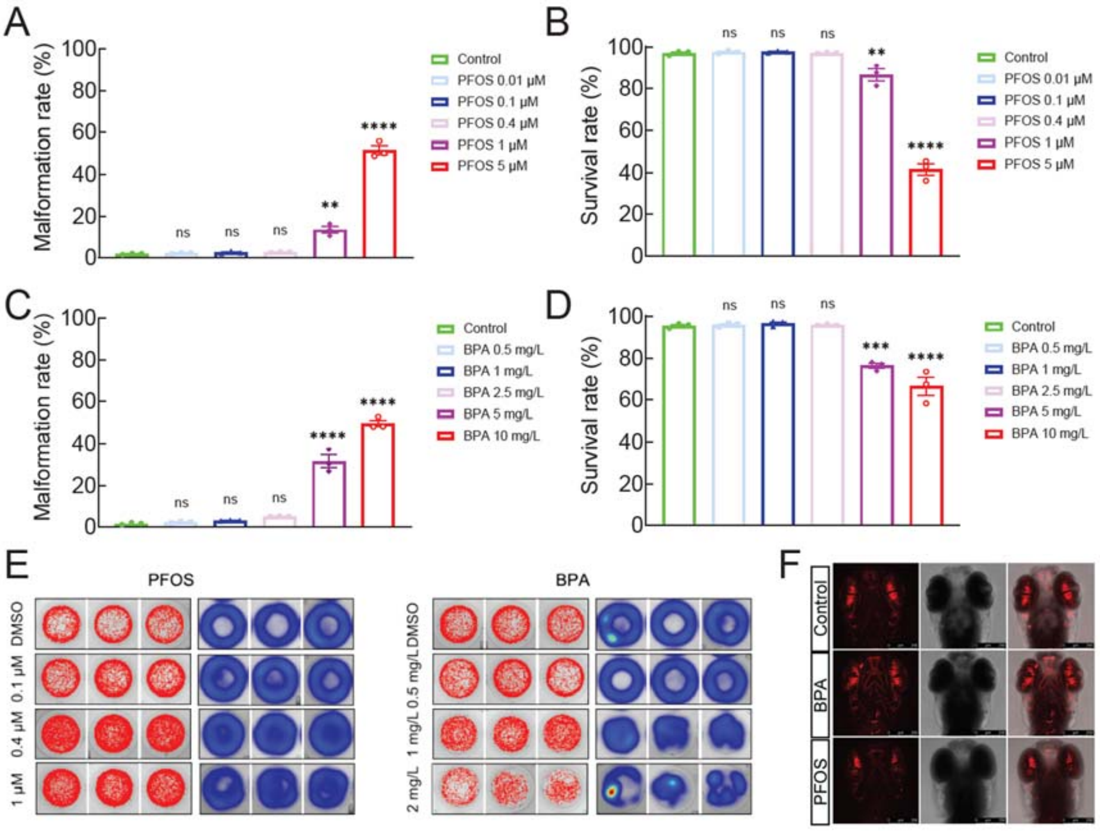

Supplementary figure S6. Related to Figure 7. (A) Malformation rate of the wild type embryos, after 3 days of PFOS exposure at the indicated concentration. (B) Survival rate of PFOS exposure at the indicated concentration. (C) Malformation rate of the wild type embryos, after 3 days of BPA exposure at the indicated concentration. (D) Survival rate of BPA exposure at the indicated concentration. (E) Swimming trajectory and heat maps of individual larvae after exposure of FFOS (left) and BPA

(right) at the indicated concentration (n=30 per group). (F) Fluorescent images showing the RFP signals in day 7 *gad1*-RPF reporter larvae that have been exposed to the indicated EEDs. Data are presented as mean ± SEM;***P* <0.01, ****P* < 0.001, *****P* < 0.0001. ns represents no significance.
